# Re-evaluating the Conceptual Framework of Health System Resilience: Insights From Economic Sanctions: Comment on "Re-evaluating Our Knowledge of Health System Resilience During COVID-19: Lessons From the First Two Years of the Pandemic"

**DOI:** 10.34172/ijhpm.8691

**Published:** 2025-06-17

**Authors:** Haniye Sadat Sajadi, Reza Majdzadeh

**Affiliations:** ^1^Knowledge Utilization Research Center, University Research and Development Center, Tehran University of Medical Sciences, Tehran, Iran.; ^2^Interdisciplinary Research and Practice Division, School of Health and Social Care, University of Essex, Colchester, UK.

**Keywords:** Health System Resilience, Resource Scarcity, Intersectionality, Health Equity, COVID-19 Response, Conceptual Framework

## Abstract

Health system resilience in the context of economic sanctions (ES) is an underexplored area. We used data from recent studies on the impact of ES on the health systems to cross-reference and assess the applicability of the conceptual framework of health system resilience (CFHSR). Reviewing the interventions implemented under ES and aligning them with the CFHSR and COVID-19 responses, we found that the CFHSR domains encompass most strategies from the ES and COVID-19 studies. However, CFHSR does not cover several strategies related to equity and teamwork. Additionally, monitoring the consequences is missing from the experiences of COVID-19 and ES. The CFHSR appears to be reasonably effective in categorizing strategies for both COVID-19 and ES. Nonetheless, its domains can be further refined. Specifically, incorporating an intersectional equity lens could enhance this conceptual framework. The next step is to develop a practical guide to apply CFHSR to strengthen health system resilience.

 In their article titled “Re-evaluating Our Knowledge of Health System Resilience During COVID-19: Lessons From the First Two Years of the Pandemic,” Saulnier and colleagues compare Blanchet and colleagues’ proposed conceptual framework of health system resilience (CFHSR) with findings from COVID-19-related studies.^1^ They suggest that current perspectives on health system resilience demonstrate a higher degree of internal consistency than in the past. The authors emphasize that we can establish connections between health system resilience and broader issues highlighted during the pandemic by considering governance. However, they also highlight areas that remain insufficiently explored, such as the short- and long-term effects of system changes and the resilience of subsystems. It underscores the need for ongoing refinement of Blanchet and colleagues’ CFHSR.

 While conceptual frameworks provide a deeper understanding and facilitate recognition of issues, they inherently lack comprehensiveness. Evaluating their utility requires real-world data to cross-referencing their effectiveness and drive further development. Saulnier and colleagues’ article plays a crucial role in enhancing our understanding of health system resilience and bridging theoretical concepts with practical strategies for building resilient health systems. Building on Saulnier and colleagues’ critical reflections, this commentary extends the analysis to another form of systemic shock: economic sanctions (ES).

 Similar to Saulnier and colleagues’ work, we applied data from our recent studies on the impact of ES on health systems^2,3^ to cross-referencing the CFHSR with real-world data. To examine how the CFHSR performs under different types of health system shocks, we specifically analyzed real-world data related to ES.

 ES are policy tools used by sanctioning agents to influence a target’s behaviour through economic restrictions, aiming to avoid military confrontation while promoting international law and human rights.^4^ Given their profound and multifaceted impacts on national health systems, ES offer a unique opportunity to evaluate the robustness and comprehensiveness of the CFHSR framework. Although designed as nonviolent measures, sanctions can severely disrupt national economies and critical sectors such as healthcare. By constraining financial resources, disrupting supply chains, and degrading public sector capacities, sanctions function as complex socio-economic shocks.^5^

 Within health systems, sanctions exacerbate existing vulnerabilities by reducing healthcare financing, restricting access to medicines and technologies, and intensifying workforce shortages.^5^ These impacts lead to increased out-of-pocket payments, shortages of essential supplies, deterioration of health infrastructure, and diminished access to quality care, disproportionately affecting marginalized and vulnerable populations.^5,6^ Indirect economic consequences—including inflation, unemployment, and currency devaluation—further undermine equitable healthcare access and weaken the resilience of health systems.^5^ Although ES represent a distinct form of exogenously imposed economic shock, their systemic impacts closely resemble those observed in other types of economic crises, such as financial bailouts or cost-of-living crises. In all these scenarios, health systems experience resource constraints, weakened governance structures, service delivery disruptions, and intensified inequalities. However, sanctions are unique in that they are politically motivated and intentionally target specific sectors, amplifying vulnerabilities in ways that may differ from endogenous economic shocks.

 ES can severely undermine the right to health by restricting access to essential medicines, healthcare services, and critical health infrastructure. As Peksen empirically demonstrates, sanctions deteriorate physical integrity rights, including access to basic health needs, and contribute to broader human rights violations.^4^ These infringements on the right to health exacerbate systemic inequalities and deepen vulnerabilities within health systems, reducing their adaptive capacity and resilience to future shocks. Thus, ensuring the protection of health rights under sanctions is not only a humanitarian obligation but also essential for maintaining the resilience of health systems in crisis-affected settings.

 Beyond their direct effects, ES generate broader political and economic constraints that critically impair adaptive capacity. Sanctioned countries often face macroeconomic instability, declining public investments, disrupted international partnerships, and deteriorating governance structures.^3^ These systemic pressures restrict the health system’s ability to reallocate resources flexibly, innovate care delivery, or maintain essential international collaborations, ultimately eroding resilience and responsiveness to new shocks.^3^

 To explore resilience strategies, we relied on two primary studies^2,3^ comprising a scoping review of global evidence and an empirical study on Iran’s health system responses to sanctions. These studies included a systematic coding process, wherein two independent reviewers extracted and categorized reported strategies according to the predefined domains of Blanchet and colleagues’ CFHSR. Coding criteria were based on the primary objectives and operational focus of each strategy. Any discrepancies between reviewers were resolved through discussion and consensus to enhance reliability. While these studies provide detailed and context-specific insights into the resilience of health systems under sanctions, they are limited to the Iranian context; therefore, caution should be exercised when generalizing the findings to broader, multi-country settings. However, this cross-referencing approach enabled a critical appraisal of the framework’s applicability in guiding real-world resilience efforts under sanctions.

 ES results from conflict between countries and directly and indirectly affect health and the health system. They can be considered a shock requiring resilience to protect population health and health equity. More specifically, ES is a diplomacy tool intended to change targeted regime behaviour without resorting to war. Although essential medicines and medical equipment are typically exempt from these sanctions, indirect impacts on health systems still affect population health.

 A study on the effect of ES on life expectancy illustrated that United Nations sanctions decrease life expectancy by 1.2-1.4 years on average, with women being disproportionately affected.^4^ A recent review found that out of 27 articles examining the effects of ES on low- and middle-income countries, 21 reported adverse health outcomes.^5^ ES disrupts health systems through various mechanisms, such as hindering financial transactions for medicines and medical equipment, reducing national revenues, and decreasing people’s ability to pay for healthcare services. To counter these shocks, countries must consider strategies to make their health systems more resilient and mitigate the impact of ES.

 For this commentary, we reviewed the strategies deployed by countries under ES, drawing exclusively from two primary studies focusing on Iran’s health system responses.^2,3^ Strategies related to COVID-19 were extracted from the review conducted by Saulnier et al.^1^ By aligning these actions with Blanchet and colleagues’ CFHSR, we developed Table, in which three groups of strategies can be distinguished: (1) Strategies considered in all three sources (CFHSR, COVID-19, and ES) and those found in either one of the two empirical data sources and CFHSR, highlighted in green; (2) Strategies found in either one of the two empirical data sources but not in CFHSR, highlighted in blue; and (3) Strategies not reported in either of the two empirical data sources but present in CFHSR, highlighted in pink.

**Table:** Differences in the Identified Aspects of Resilience Against Economic Sanctions, COVID-19, and the Conceptual Framework of Health System Resilience

**Domains**	**Strategies Extracted From ES Studies**^a^	**ES**	**COVID-19**	**CFHSR**
Learning	1. Preparedness and planning for sanctions	*	*	*
Knowledge sharing	2. Assessing the effects of policies and international agreements on the health of people 3. Determining the exact magnitude of the impact of sanction 4. Developing an integrated information system for monitoring the market 5. Employing cost-effectiveness evidence for pricing and reimbursement 6. Establishing and improving a robust surveillance system. 7. Institutionalizing economic evaluation of medicines, medical devices, equipment, and health services 8. Tracking medication 9. Strengthening evidence-informed policy-making 10. Strengthening health monitoring systems	*	*	*
Leadership	11. Revising health management 12. Strong leadership and management 13. Boosting the morale, knowledge, skills, and innovation of managers to potentially increase resilience	*	*	*
Uncertainties	14. Adapting exportation laws based on domestic needs 15. Allocating a protected SWIFT line specifically for humanitarian medicines trade 16. Amending the OFAC EAR99^b^ classification system to make it easier for companies to export medicines 17. Centralized and strategic purchasing 18. Considering collateral pathways for procurement of required medical items	*	*	*
Uncertainties (*continue*)	19. Creating efficient food assistance programs by the government and the international community, funding food banks with the assistance of charities and non-governmental organizations, and involving individuals in nutritional education programs to learn how to plan a cheap and balanced diet 20. Developing policies and laws to alleviate the negative impacts of agreements on the human rights of the population 21. Developing the national essential medicines list 22. Implementing electronic health records 23. Implementing electronic prescriptions 24. Establishing regulatory export harmonization 25. Establishing uniform criteria and definitions for exemptions as well as operational criteria for sanctions committees to facilitate improved effectiveness of exemptions 26. Exempting medicine and medical commodities from “snap back” provisions 27. Exempting vaccine products from stringent export controls 28. Facilitating immediate release of medicines, food, and medical equipment from customs with minimal financial documents. Funding health via sustained sources 29. Funding health via sustained sources 30. Improving the system for fair and effective allocation of resources between health plans and relevant executive bodies in health 31. Institutionalizing fair and effective resource allocations 32. Investing in domestic production 33. Managing and developing health tourism 34. Mobilizing latent resources in education and health 35. Optimizing the use of human resources (by improving competencies and making appropriate use of job descriptions, eg, avoiding specialization in basic services) 36. Reducing the price of imported medicines through public resources. 37. Implementing proactive inventory control 38. Proper implementation of the referral system 39. Providing a national policy with measures to prevent the suffering of people from the adverse effects of sanctions	*	*	*
Uncertainties (*continue*)	40. Providing additional clarification that main sources of revenues can be freely used for medicine procurement without reservations 41. Providing clinical guidelines for rational prescribing 42. Rationalizing the prices of medicines 43. Refocusing health policy toward maximizing scarce resources 44. Strengthening the insurance system 45. Supporting local production 46. Using alternative medicines and methods 47. Using the capacity of some international intermediate organizations and certain companies and financial institutions to facilitate purchasing medical items	*	*	*
Interdependence	48. Preventing third parties, black market dealers, pharmacies, and health facilities from providing unsafe medicines, as well as stopping smugglers 49. Refraining from imposing embargoes and other measures that restrict the supply of medicine and medical equipment	*	*	*
Legitimacy	50. Creating mutual trust among different organizations 51. Deploying international intermediate organizations, companies and financial institutions to facilitate the implementation of exemptions 52. Determining a memorandum of understanding between the Iran’s Food and Drug Administration, health insurance organizations, and the central bank 53. Empowering the community and increasing their participation 54. Establishing an appropriate organizational structure to deal with sanctions 55. Informing the medical community 56. Mobilizing public participation to compensate for reduced access to capital goods 57. Paying more attention to mass media 58. Professional organizations, especially those concerned with children's health, must advocate for children in countries experiencing ES	*	*	*
Monitor risks from beyond the health system	59. Observing the situation of human rights, implementing humanitarian and human rights laws	*		*
Vertical interdependence within the health system	60. Maintaining constant collaboration and active social networks at both national and global levels 61. Strengthening inter-sectoral cooperation	*		*
Teamwork	62. Strengthening global health diplomacy 63. Consumer–patient collaboration 64. Enhancing interactions with neighboring countries 65. Using all available political and legal means, such as health diplomacy, to establish humanitarian channels, enhance global conventions, and remove possible barriers like sanctions to reduce their adverse consequences on antimicrobial resistance control	*	*	
Health system actor legitimacy			*	
Values	66. Emphasizing preventative over curative medicine	*	*	
Equity	67. Developing dual policies of equity and priority for vulnerable groups 68. Establishing support mechanisms to prevent and control the social harms of the economic outcomes of sanctions (eg, the protection of working children) 69. Providing extra financial protection for special, incurable, and chronic patients and allocating additional budget to over-compensate for unaffordable pharmaceutical products 70. Protecting vulnerable groups of the population, such as children and the poor 71. Ensuring rationing, universal access to primary health care, a highly educated population, and preferential access to scarce goods for women and children 72. Using public systems to motivate behavioral change, with a focus on the needs of women and children 73. Defining tailored health service packages for vulnerable populations	*	*	
Transsectoral			*	
Private sector	74. Delegation and privatization	*	*	
Short- and long-term consequences of the changes on the system^c^				*

Abbreviations: CFHSR, Conceptual Framework of Health System Resilience; ES, economic sanctions; OFAC, Office of Foreign Assets Control; EAR, Export Administration Regulations. ^a^ Key components of this column are adapted from the author’s earlier work.^3^ Appropriate attribution has been provided to avoid redundancy and uphold academic integrity. ^b^ Under US export control law, EAR99 designates items that fall under the EAR but are not listed on the Commerce Control List. Such items typically do not need an export license for most destinations. However, when the recipient country is under US sanctions, like Iran, separate authorization from the OFAC is still required—even for EAR99-classified goods, including many standard pharmaceuticals. ^c^ In the domains where corresponding strategies were not present in the studies related to ES, the cells corresponding to the strategies have been left blank.

 In the first group, a series of strategies are observed in all three sources. This indicates that the four aspects mentioned in the framework seem suitable for understanding how to enhance system resilience. Two noteworthy points:

 Firstly, the high number of ES resilience strategies categorized under the domain of uncertainties. These strategies mainly focus on facilitating financial transactions, resilience against financial resources’ constraints, self-reliance on domestic resources, developing focused plans and policies to respond to ES, and re-organizing and reforming health service delivery. The large number of these strategies in this category indicates that uncertainties accompany conditions in ES. When facing ES, the top priority is to address how the financial resources’ constraints could be managed. The predominance of strategies within the uncertainties domain likely reflects the acute operational challenges posed by ES. Sanctions primarily create unpredictability in resource availability, governance stability, and international transactions, forcing health systems to prioritize flexible, immediate responses to manage financial constraints and supply disruptions. In contrast, fewer strategies were identified in financing and human resources domains, possibly because structural reforms in these areas require long-term investments and stable environments, which are difficult to achieve during acute sanction crises.^2^

 Among the three dimensions, transformative capacity has received the least attention relative to absorptive and adaptive capacities. As mentioned, ES immediately impact resource limitations and access to medicine and equipment, both of which are crucial for the essential functions of a health system.^6^ Therefore, policy-makers are seeking immediate solutions to resolve issues related to resource issues. However, it is crucial not to let adaptive solutions overshadow transformative actions, and significant importance should be given to the latter. For the health system to be resilient, especially in the long term, it is essential to consider transformative strategies alongside addressing resource issues and institutionalizing the health system to reduce vulnerability based on lessons learned.^7,8^

 Secondly, regarding the CFHSR, while the framework effectively categorizes broad domains relevant to health system resilience, it does not clearly delineate the contribution of each strategy to the specific phases of resilience—absorption, adaptation, and transformation. This lack of phase-specific guidance may limit the framework’s operational utility, particularly during complex shocks such as ES, where different types of responses must be prioritized at different stages. More explicit mapping of domains like knowledge sharing, uncertainty, legitimacy, and interdependency across resilience phases could significantly enhance the framework’s practical applicability and strategic value. Addressing these limitations could substantially enhance the CFHSR’s utility in guiding resilience-building efforts under complex, protracted crises.

 Another part of the strategies in this group pertains to those shown in the CFHSR and present in one of the two data sources (ES or COVID-19). Therefore, there is empirical evidence supporting these domains of the CFHSR. As indicated in Table at the end of the sections highlighted in green, “Monitor risks from beyond the health system” and “Vertical interdependence within the health system” comprise this section.

 In the context of ES, one serious consideration is human rights aspects. The United Nations Human Rights Council has frequently assessed the human rights aspects of ES in various countries and reported its impact on Sustainable Development Goals.^9^ The most effective preventive intervention in this regard is likely to use international law capacities to influence sanction regimes and protect people from their risks. The second domain pertains to forming international and national coalitions that undertake protective measures against ES. Fortunately, both of these are considered in the CFHSR.

 The second group, highlighted in blue in Table, is the most important one, where the potential gaps in CFHSR can be identified. This group includes strategies in equity, teamwork, health system actor legitimacy, values, trans sectoral approaches, and the private sector.

 The majority of strategies relate to equity. Evidence shows that ES can affect health and the living standards of individuals in society.^9^ Thus, protecting vulnerable groups, especially those facing the intersection of gender, age, informal work, and other sources of oppression in the community, is crucial and must be seriously considered in the CFHSR, which is a substantial shortfall in the conceptual framework of community resilience against ES. The intersectionality approach is vital for promoting equity in resilience by pinpointing and advocating for groups vulnerable to health insecurity.^10^ We stress that intersectionality is essential for tackling health disparities, especially in marginalized communities during health system disruptions, to ensure that no one is left behind. We contend that effective and comprehensive resilience strategies must account for the intricate interactions of individuals’ diverse identities with various power structures, necessitating integrated responses suited to different contexts. Overlooking the complexity of intersectionality may lead to interventions that fall short in meeting the varied needs of different population groups.

 The COVID-19 articles include two domains, teamwork and the private sector, which have also been observed in articles related to ES. These collaborations are crucial in the health system’s resilience to utilize all possible national (such as the private sector) and international (regional and beyond cooperations) efforts to meet the needs for medicine, equipment, and capacity building. For COVID-19, these collaborations provided services (private sector), COVID-19 vaccine supply, and equipment (preventive or therapeutic). At the same time, ES encompasses a broader range, including the provision of raw materials, medicine, services, financial interactions, and investment in health infrastructure. Between the two areas discussed—using an intersectional lens to avoid inequities and developing national and international coalitions—the former is relevant to almost all cases of health system resilience. However, the latter may vary depending on the circumstances.

 Another central issue in the ES articles emphasized primary prevention and primary health care over more costly and specialized diagnostic and therapeutic services. Due to financial resource constraints, promoting economic resistance programs is suggested, and emphasizing such services to avoid more expensive and less efficient interventions is seen as a strategy of “Value.” These aspects also deserve consideration in the CFHSR.

 Two other domains that we did not encounter in the ES articles but are already addressed in the article by Saulnier and colleagues are health system actor legitimacy and transsectoral approaches, and we avoid elaborating on these in this commentary.

 Finally, the third group includes a single strategy: short- and long-term consequences of the changes on the system, which is present in CFHSR but not seen in the other two empirical data sources. If the crisis is short-term, it is challenging to assess these effects. As soon as ES is imposed, changes occur, some of which are very rapid and noticeable, such as the shortage or increased cost of medicine, vaccines, and medical equipment. In the field of research, there are also restrictions on access to databases. These issues usually become priorities for response. However, monitoring the more implicit and long-term consequences becomes challenging, such as impacts on social capital, quality of life, quality of services, migration, and the isolation of the health sector. Additionally, there are consequences for social and economic conditions, which gradually affect health. The complexity of the mechanisms by which ES impact health has been noted in previous research.^2^ Even when interventions are implemented, assessing their effectiveness is difficult. Therefore, while acknowledging that this is one of the most critical aspects to consider for the health system’s resilience, it is practically challenging and needs to be studied more scientifically and systematically.

 In conclusion, CFHSR is reasonably effective in organizing and categorizing strategies for COVID-19 and ES health system resilience. The present commentary indicates room for improvement in areas such as focusing on strategies that adopt an intersectional approach to equity and support marginalized groups in the community, as well as in comprehensive collaborations and utilizing all national and international capacities (such as international laws). Additionally, the contribution of strategies in the three phases of absorb, adapt, and transform can be clarified.

 Creating a user-friendly guide would help policy-makers leverage this framework more effectively in real-world situations, ensuring timely and robust responses to health system shocks. By strengthening both conceptual frameworks and practical tools, health systems can be better equipped to protect population health amid political and economic adversity.

## Ethical issues

 Not applicable.

## Conflicts of interest

 Authors declare that they have no conflicts of interest.
